# N-Terminus Three Residues Deletion Mutant of Human Beta-Defensin 3 with Remarkably Enhanced Salt-Resistance

**DOI:** 10.1371/journal.pone.0117913

**Published:** 2015-02-23

**Authors:** Tao Li, Feng Guo, Qin Wang, Huali Fang, Zhan Li, Dehui Wang, Hui Wang

**Affiliations:** State Key Laboratory of Pathogens and Biosecurity, Beijing Institute of Microbiology and Epidemiology, Fengtai District, Beijing, PR China; Universidad Nacional de La Plata., ARGENTINA

## Abstract

In this study, we designed and synthesized three N-terminal deletion analogs of human beta-defensin 3 (hBD-3), namely, hBD-3Δ4, hBD-3Δ7, and hBD-3Δ10, to determine the effect of N-terminal residues on the antibacterial activity and salt resistance of these peptides. The antibacterial activities and salt resistance of hBD-3 and its analogs were tested against a broad range of standard and clinically isolated strains. The deletion of nine N-terminal residues significantly reduced the antibacterial activity of hBD-3 against most of tested strains, particularly *Klebsiella pneumonia*. Compared with hBD-3 and other analogs, the analog with a deletion of three residues, hBD-3Δ4, exhibited significantly higher antimicrobial activity against almost all the tested strains, especially *Escherichia coli* and *Enterococcus faecium*, at high NaCl concentrations. Given its broad spectrum of antimicrobial activity and high salt resistance, hBD-3Δ4 could serve as a promising template for new therapeutic antimicrobial agents.

## Introduction

Human defensins, which are rich in cationic amino acids and cysteine residues, are crucial components of the innate immunity of humans [1, 2]. The broad antibacterial spectrum and high antibacterial activity of human defensins make them important effector molecules of mucosal surface, skin, and epithelia [3]. Similar to other antibacterial peptides, such as indolicidins, gramicidins, bactenecins, and magainins, defensins are salt sensitive [4]. The disturbance of electrostatic interactions may reduce the activity of antimicrobial peptides [5].

Human beta-defensins 1 to 4 (hBD-1 to hBD-4) exert different bactericidal activities against various pathogens [1–3]. Compared with hBD-1 [6], hBD-2 [7], and hBD-4 [8], human beta-defensin 3 can withstand higher NaCl concentrations because of its irregular structural characteristics and charge [9]. hBD-3 is a 45-residue cationic peptide with an asymmetrical distribution of charged residues clustered mostly at the carboxyl-terminal (C-terminal) region [10]. The nuclear magnetic resonance solution structure of hBD-3 shows three anti-parallel beta-sheets and a short helical loop at the N-terminal region [11]. The 3D structure of hBD-3 is different from those of other hBDs. In particular, the N-terminal helix of hBD3 starts from Lys8, whereas residues 1 to 7 are disordered. Thus, the effect of N-terminal residues on the antibacterial activity and salt resistance of hBD-3 must be systematically determined.

In this study, we designed and synthesized three N-terminal deletion analogs of hBD-3, which in principle would be similar to hBD-3 in terms of antibacterial activity and high NaCl resistance. We then compared the antibacterial activities of the novel synthetic analogs with those of hBD-3. Results showed that one of the synthetic analogs had significantly higher antimicrobial activity than the hBD-3 at high NaCl concentrations.

## Materials and Methods

### 1. Bacterial strains and growth conditions

Ten strains obtained from the American Type Culture Collection (ATCC) and China Center of Industrial Culture Collection (CICC) were used for antibacterial and salt resistance assays. The Gram-positive strains were *Staphyloccocus aureus* ATCC 29213, *Enterococcus faecalis* ATCC 29212, *Staphylococcus epidermidis* ATCC 12228, *Enterococcus faecium* ATCC 6057, and *Enterococcus faecium* ATCC 19434. The Gram-negative strains were *Escherichia coli* ATCC 25922, *Pseudomonas aeruginosa* ATCC 15442, *Klebsiella pneumonia* ATCC 700603, *Shigella flexneri* CICC 21534, and *Shigella sonnei* CICC 21535. Fifteen *E. coli* clinical isolates were collected from 302 Military Hospital of China. The Gram-positive and Gram-negative strains were grown in Luria—Bertani (LB) and Mueller—Hinton (MH) media at 37°C, respectively.

### 2. Peptide synthesis and analysis

Peptides (all sequences shown in Table 1) hBD-3 (full-length hBD3 without native disulfide pairings), hBD-3Δ4 (residues 4 to 45 of hBD3 without native disulfide pairings), hBD-3Δ7 (residues 7 to 45 of hBD3 without native disulfide pairings), and hBD-3Δ10 (residues 10 to 45 of hBD3 without native disulfide pairings) were synthesized by the standard solid-phase 9-fluorenylmethoxycarbonyl method [12] and then dissolved in ddH_2_O for all experiments. The identity and purity of all peptides were confirmed by electrospray ionization-mass spectrometry and reversed phase-high-performance liquid chromatography. Circular dichroism (CD) spectra were recorded with a Jasco-J810 in a 2 mm quartz cell at room temperature. The spectra of hBD-3 and its analogs were the average of three consecutive scans from 260 nm to 190 nm, recorded with a data pitch of 0.5 nm.

**Table 1:** Sequences and net charges of hBD-3, and N-terminus deletion analogs.

Peptide	Sequence	Length (amino acids)	Net charge
hBD-3	GIINTLQKYYCRVRGGRCAVLSCLPKEEQIGKCSTRGRKCCRRKK	45	11
hBD-3Δ4	NTLQKYYCRVRGGRCAVLSCLPKEEQIGKCSTRGRKCCRRKK	42	11
hBD-3Δ7	QKYYCRVRGGRCAVLSCLPKEEQIGKCSTRGRKCCRRKK	39	11
hBD-3Δ10	YCRVRGGRCAVLSCLPKEEQIGKCSTRGRKCCRRKK	36	10

### 3. Antibacterial activity and salt resistance assay

The antibacterial activities of these peptides were determined in a modified microdilution assay as described previously [12]. The strains were grown under aerobic condition in MH or LB broth at 37°C and then harvested in the exponential phase of growth (OD_600_ ≈ 0.6. OD, optical density). After dilution, the test strains were adjusted to 10^5^ to 10^6^ CFU bacteria per ml in phosphate-buffered saline solution (pH 7.2). The five concentrations of the peptides were added to 200 μl of the bacterial suspension and then incubated at 37°C for 3 h before CFU numbers were determined. Phosphate buffer served as the negative control. The cells were serially diluted in the same buffer, plated on Luria broth plates, and then incubated for 18 h at 37°C before the resulting colonies were counted. The bactericidal activity was expressed as the ratio of colonies counted to the number of colonies on a control plate. The 90% lethal concentration (LD_90_) is the concentration of the peptide at which 90% of viable cells are killed.

For the salt resistance assay, NaCl was included in the incubation buffer (1% MH or LB) over a range of concentrations (0, 50, 100, 150, and 200 mM NaCl) potentially present in the airway surface fluid of healthy subjects and patients with cystic fibrosis [13]. For *E. coli* clinical isolates, the NaCl concentrations were increased to 0, 50, 100, 150, 200, 250, and 300 mM. The peptide concentration used in the salt dependence assay was 10 μg/ml, except for the 30 μg/ml concentration used against *K. pneumonia* ATCC 700603. Each assay was performed in triplicate. Bactericidal activity (the mean and standard deviation of three assays) was expressed as the ratio between the number of colonies counted and the number of colonies on a control plate.

### 4. Cytotoxicity

Vero cells suspended in Dulbecco’s modified Eagle’s medium (DMEM)-high glucose culture medium supplemented with 10% (vol/vol) fetal calf serum and 1% (vol/vol) penicillin—streptomycin were seeded on a 96-well microtiter plate at a density of 1×10^4^ cells per well. After incubation overnight at 37°C under 5% CO_2_ in a humidified atmosphere, the cells were washed twice with buffer (pH 7.4) containing 20 mM HEPES and 150 mM NaCl. Defensins were diluted in the same wash buffer, and 200 μl was added per well. The buffer was discarded after 2 h of incubation at 37°C under 5% CO_2_. Subsequently, 200 μl of DMEM and 50 μl of 3-(4,5-dimethylthiazol-2-yl)-2,5-diphenyltetrazolium bromide (MTT, 5 mg/ml) were added. DMEM (100%) and MTT were discarded, and 200 μl of DMSO was added in the plate after 6 h of incubation at 37°C under 5% CO_2_. The absorbance was measured at 490 nm with a microtiter plate reader.

### 5. Statistical methods

All data were summarized by using the mean and standard deviation computed for three independent replicates. Differences in means were tested by independent samples t—test or non parametric test.

## Results

### 1. Antibacterial activities of hBD-3 and its N-terminal deletion analogs

The effect of N-terminal residues on the antibacterial activities of hBD-3 and its three N-terminal deletion analogs (hBD-3Δ4, hBD-3Δ7, and hBD-3Δ10) was determined. The sequences of hBD-3 and its analogs are shown in Table 1. hBD-3 contains all amino acids, whereas hBD-3Δ4, hBD-3Δ7, and hBD-3Δ10 only contain residues 4 to 45, residues 7 to 45, and residues 10 to 45 of hBD-3, respectively. The synthesized peptides have the same net charge (+11), except for hBD-3Δ10 with a +10 net charge.

The susceptibility of these peptides against a broad range of human Gram-negative and Gram-positive pathogenic bacteria was tested. The LD_90_ values of hBD3 and its analogs were determined by conventional broth micro-dilution assays. Table 2 lists the antibacterial activities of the synthetic peptides against the 10 tested strains. Peptides hBD3, hBD-3Δ4, and hBD-3Δ7 exhibited strong antibacterial activity against all the tested bacteria, except for *K. pneumonia* ATCC 700603. The LD_90_ values of these analogs ranged from 1.6 to 8.0 μg/ml. Compared with hBD-3 and the other analogs, hBD-3Δ10 showed a significantly weaker antibacterial activity against most of the tested bacteria, except for *S. aureus* and *E. coli* (P ≤ 0.01). This result showed that the deletion of more than nine N-terminal amino acids affected the antibacterial activities of hBD-3. However, the LD_90_ values of hBD3, hBD-3Δ7, and hBD-3Δ10 against *K. pneumonia* were significantly higher than against the other tested bacteria. Particularly, the LD_90_ of hBD-3Δ10 reached more than 100 μg/ml. Generally, hBD-3Δ4 showed significantly higher antibacterial activity than the other synthesized peptides.

**Table 2:** Anti-bacteria activity of hBD-3 and its analogs.

Peptide	LD_90_ (Mean ± SD, μg/ml)				
	*S. aureus* ATCC 29213	*E. faecalis* ATCC 29212	*S. epidermidis* ATCC 12228	*E. faecium* ATCC 6057	*E. faecium* ATCC 19434
hBD-3	4.0±0.8	2.4±1.0	3.5±1.2	1.6±0.4	2.3±0.5
hBD-3Δ4	2.5±0.6	2.6±0.3	6.5±0.7	3.4±0.4	2.6±0.2
hBD-3Δ7	1.6±0.2	1.9±0.4	4.0±1.0	2.0±0.6	2.1±0.4
hBD-3Δ10	3.8±2.0	5.0±1.4 **	7.0±1.3 **	5.0±1.2 **	6.4±0.7 **

Peptide	LD_90_ (Mean ± SD, μg/ml)				
	*E. coli* ATCC 25922	*P. Aeruginosa* ATCC 15442	*K. pneumonia* ATCC 700603	*S. flexneri* CICC 21534	*S. sonnei* CICC 21535
hBD-3	8.0±1.2	6.8±0.9	18.6±2.8	3.7±0.2	4.0±0.7
hBD-3Δ4	4.6±1.0	6.1±1.2	7.5±1.7	3.3±0.4	3.2±0.3
hBD-3Δ7	6.6±0.8	6.0±1.2	28.0±4.0	2.6±0.4	3.0±0.4
hBD-3Δ10	10.0±3.4	13.5±3.2 **	>100 **	15.3±1.8 **	20.6±3.2 **

** Significantly different (*P* < 0.01) from the activity of wild-type hBD-3.

### 2. Salt resistance of hBD-3 and its analogs

**Fig 1:**
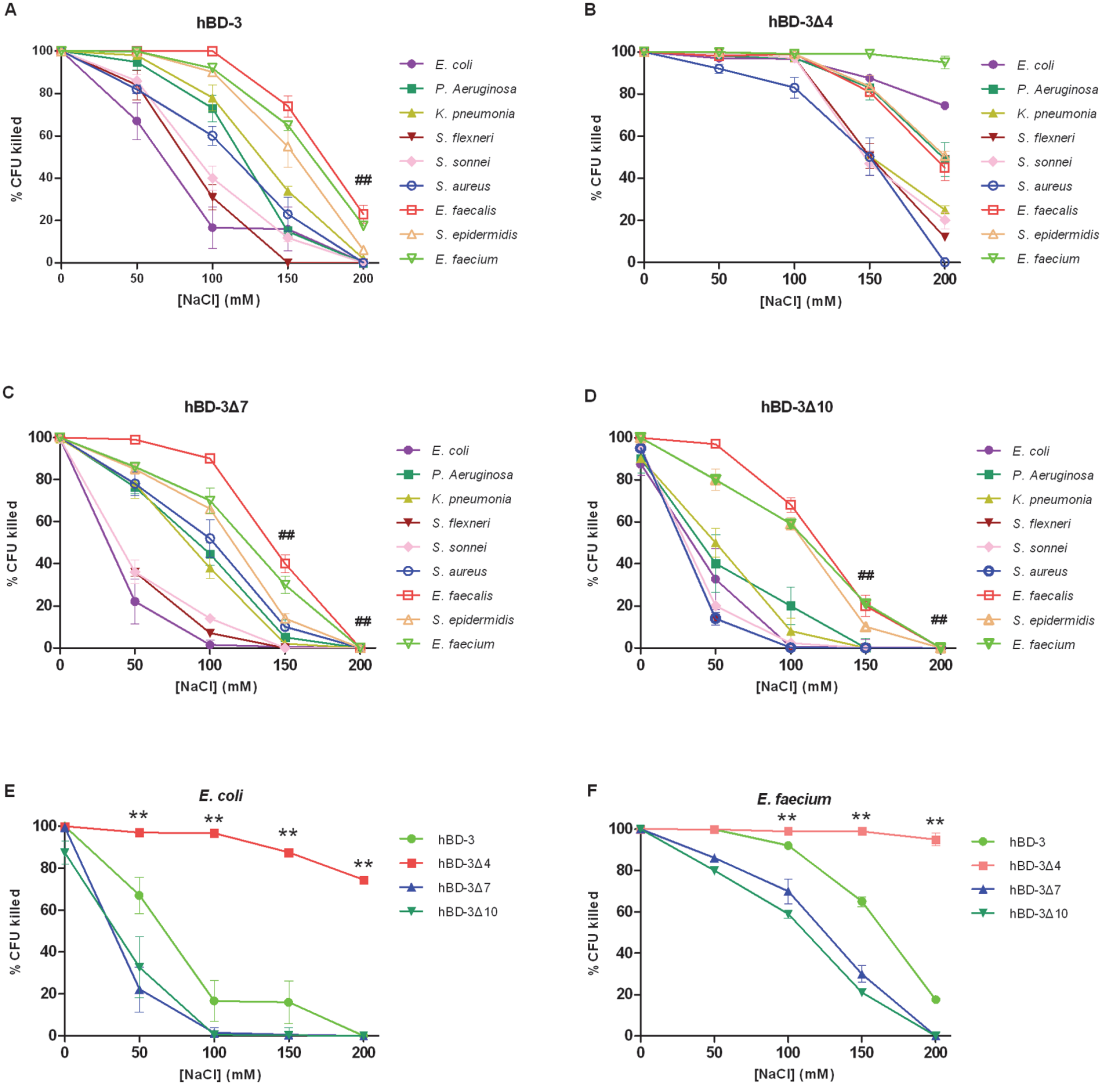
Antibacterial activity at increasing NaCl concentrations of hBD-3 (A), hBD-3Δ4 (B), hBD-3Δ7 (C) and hBD-3Δ10 (D) against nine bacterial strains or species; and of hBD-3 and analogs against E. coli ATCC 25922 (E) and *E. faecium* ATCC 6057 (F). Error bars show the SDs of experiments performed in triplicate. Non parametric tests were used in the statistical analysis. ** Significantly different (*P* < 0.01) from the activity of wild-type hBD-3. ^##^ Significantly different (*P* < 0.01) from the activity of hBD-3 at 0 mM NaCl.

The antibacterial activities of hBD-3 and its N-terminal deletion analogs against four Gram-positive and five Gram-negative bacteria, expressed as the percentage of CFU killed, were evaluated to determine their salt resistance (Fig. 1). The concentration of each peptide (10 μg/ml except with 30 μg/ml peptides against *K. pneumonia* ATCC 700603) and five concentrations of NaCl (0, 50, 100, 150, and 200 mM) were used.

The activity of hBD-3 against all the tested strains, except for *E. coli*, was not or slightly inhibited by 50 mM NaCl (Fig. 1A). The antimicrobial activity of hBD-3 against the different bacteria varied with 100 or 150 mM NaCl. At 100 mM NaCl, hBD-3 killed more than 90% CFU of *E. faecalis*, *S. epidermidis*, and *E. faecium* as well as 60% to 80% CFU of *P. Aeruginosa*, *K. pneumonia*, and *S. aureus*. By contrast, hBD-3 killed below 40% CFU of *E. coli*, *S. flexneri*, and *S. sonnei*. The activity of hBD-3 was greatly inhibited by 200 mM NaCl (paired *t* test versus the result for hBD-3 activity at 0 mM NaCl, *P* < 0.01).

The antibacterial activity of hBD-3Δ7 against the tested species reduced at high NaCl concentrations (Fig. 1C). The analog hBD3Δ7 was similar to hBD3 in terms of antibacterial activity against most of the tested strains (Fig. 1C) but demonstrated weaker salt resistance. At 100 mM NaCl, hBD3Δ7 killed more than 90% of *E. faecalis*, 66% and 70% CFU of *S. epidermidis* and *E. faecium*, respectively, and 38% to 52% CFU of *P. Aeruginosa*, *K. pneumonia*, and *S. aureus*, but only below 15% CFU of *E. coli*, *S. flexneri*, and *S. sonnei*. hBD-3Δ10 showed the same salt resistance as hBD3Δ7 in all tested strains but exhibited significantly weaker antibacterial activity at high NaCl concentrations (Fig. 1D).

Compared with hBD-3 and the other analogs, hBD-3Δ4 exhibited a significantly higher antimicrobial activity against almost all the tested strains at high NaCl concentrations (Fig. 1). At 100 mM NaCl, hBD-3Δ4 depicted almost the same antimicrobial activity as it would have in the absence of NaCl against all the tested strains, except for *S. aureus* (Fig. 1B). In the case of *E. coli*, hBD-3Δ4 was less sensitive to high NaCl concentrations and maintained a considerably decreased but still remarkable activity (87.5% CFU killed) in the presence of 150 mM NaCl, whereas hBD-3 was almost inactive (16.0% CFU killed) (Fig. 1E). The activity of hBD-3Δ4 was slightly inhibited (74.5% CFU killed) by 200 mM NaCl. In the case of *E. faecium*, hBD-3Δ4 showed a similar high antimicrobial activity with high NaCl concentrations, having killed 95% CFU at 200 mM NaCl (Fig. 1F).

In summary, the antimicrobial activities of hBD-3Δ7 and hBD-3Δ10 were more salt sensitive than that of hBD-3 against both Gram positive and Gram negative strains. The analog hBD-3Δ4, with a deletion of three residues, was more salt resistant than hBD-3, especially for *E. coli* and *E. faecium* (Fig. 1).

### 3. Antibacterial activities of hBD-3 and hBD-3Δ4 with high NaCl concentrations against clinical isolates

**Fig 2:**
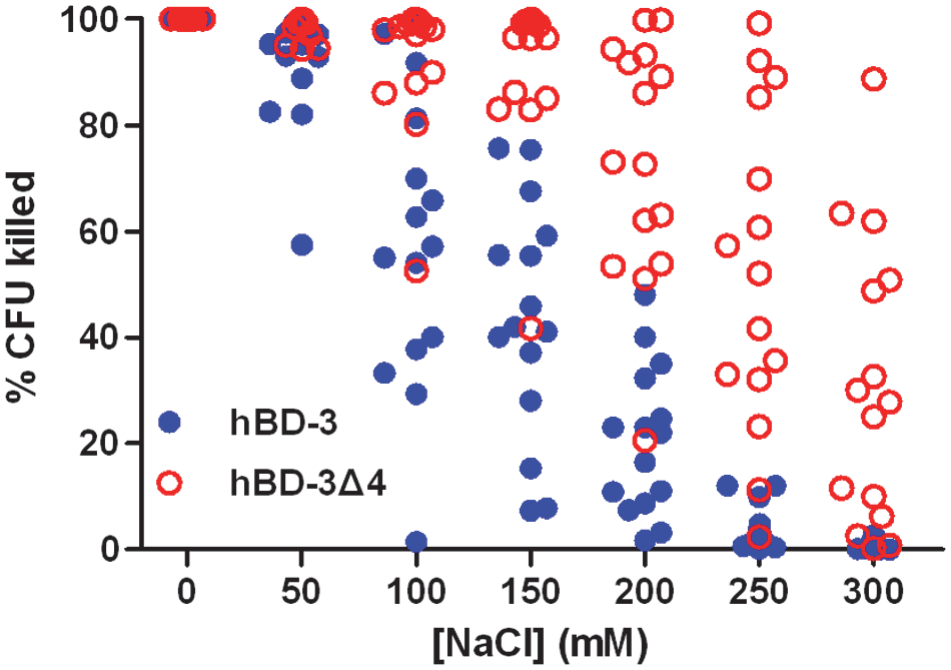
Antibacterial activity at increasing NaCl concentrations of hBD-3 and hBD-3Δ4 against fifteen *E. coli* clinical isolates.

Given the exceptional antimicrobial activity and remarkable salt tolerance of hBD-3Δ4, we tested 15 *E. coli* clinical isolates for their susceptibility to this peptide at seven NaCl concentrations (0, 50, 100, 150, 200, 250, and 300 mM) (Fig. 2, Table 3). A similar trend of salt resistance of hBD-3Δ4 was detected in the clinical isolates as *E. coli* ATCC 29212. Compared with hBD-3, hBD-3Δ4 exhibited a significantly higher antimicrobial activity at high NaCl concentrations, especially ≥ 100 mM (*P* < 0.01, Fig. 2, Table 3). At 150 mM NaCl, hBD-3Δ4 killed 91.1% ± 15.6% CFU, whereas hBD3 only killed 44.6% ± 22.1% CFU. hBD-3Δ4 retained more than 50% activity in 14, 8, and 5 isolates at 200, 250, and 300 mM NaCl, respectively. By contrast, hBD-3 retained 50% activity in two isolates at 200 mM NaCl and 0% activity at 250 or 300 mM NaCl.

**Table 3:** Anti-bacteria activities of hBD-3 and hBD-3Δ4 against fifteen *E. coli* clinical isolates at different NaCl concentrations.

Peptide	% Killed CFU (Mean ± SD) at NaCl concentrations of						
	0mM	50mM	100mM	150mM	200mM	250mM	300mM
hBD3	100	92.3±11.1	60.5±2.7	44.6±22.1	22.0±13.9	4.8±4.2	1.3±0.1
hBD-3Δ4	100	98.6±2.2	92.7±12.6 **	91.1±15.6 **	74.7±22.8 **	53.7±30.3 **	32.1±0.3 **

** Significantly different (*P* < 0.01) from the activity of wild-type hBD-3.

### 4. The CD spectroscopy of hBD-3 and its three N-terminal deletion analogs

**Fig 3:**
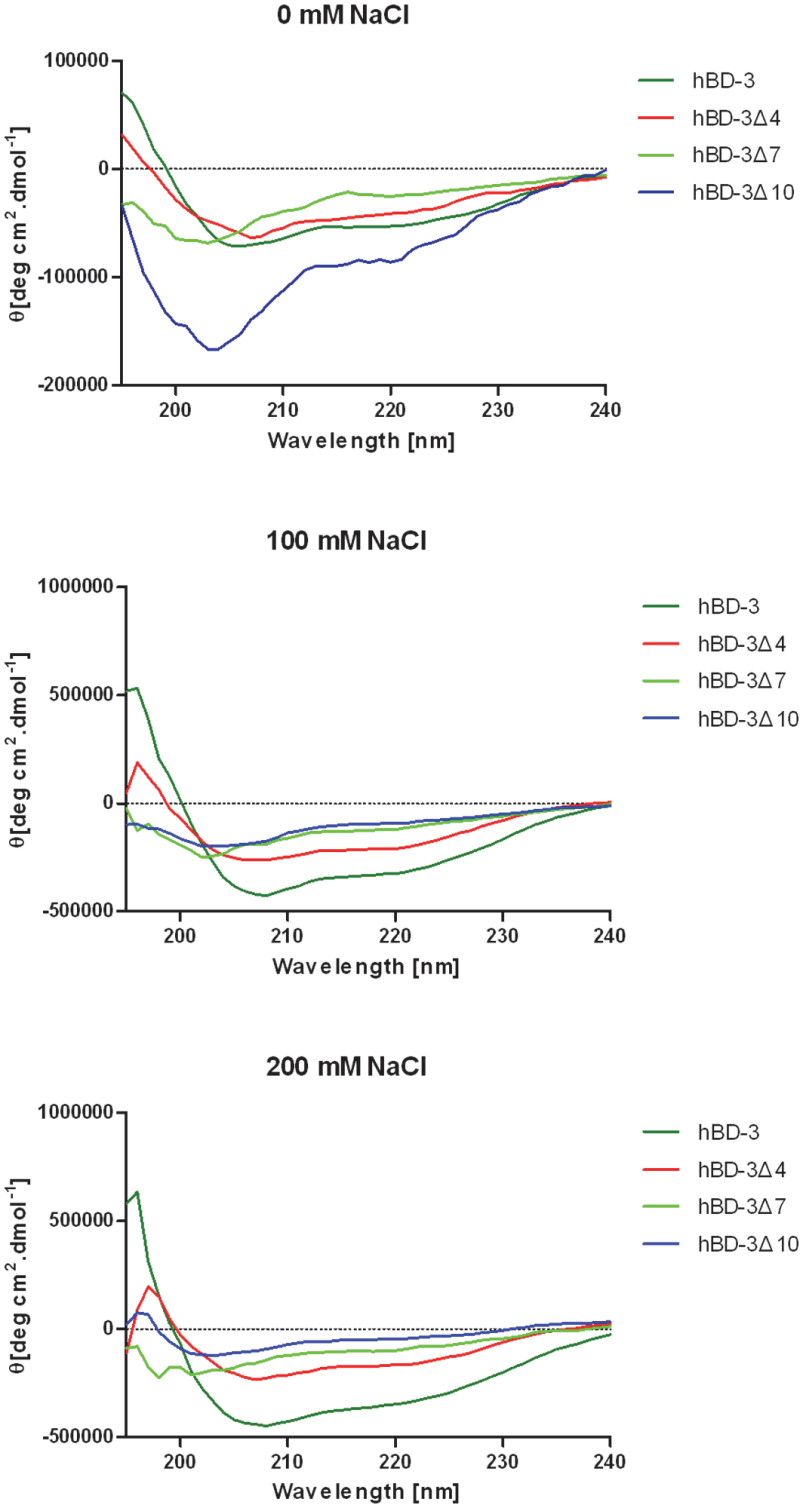
The circular dichroism spectroscopy of hBD-3, hBD-3Δ4 and hBD-3Δ7 with different NaCl concentrations.

The secondary structures of hBD-3 and its three N-terminal deletion analogs with different NaCl concentrations were analyzed by Circular dichroism spectra (Fig. 3). Their CD spectra of hBD-3 and hBD-3Δ4 with different NaCl concentrations showed clearly observable double minima at wavelengths of 209 and 222 nm, and strong positive peaks at 190–193 nm. Such a spectrum is characteristic of a-helical structures and the CD spectrum of wild-type hBD-3 agreed very well with previous reported [14]. But the CD spectra of hBD-3Δ7 and hBD-3Δ10 did not show obvious α-helix structures. And a big change in CD spectrum of hBD-3Δ10 when exposed to increasing concentration of NaCl, whereas the other deletion analogs show more conserved CD spectra with lower peaks at 0 NaCl concentration compared to hBD-3Δ10.

### 5. Cytotoxicity

hBD-3Δ4 is a promising candidate for therapeutic use because of its capacity to retain high antibacterial activity even at high NaCl concentrations. Together with hBD-3 and the other analogs, hBD-3Δ4 was tested for its cytotoxic effect on nucleated, metabolically active human cells. The vero cell line was used as a model. hBD-3Δ4 showed no cytotoxicity at 10 μg/ml (Table 4) but reached 45% cytotoxicity at 20 μg/ml. By contrast, hBD-3Δ10 did not show any significant cytotoxic effect, even at 60 μg/ml. hBD-3 demonstrated cytotoxicity against vero cells starting from a concentration higher than 20 μg/ml, reaching 48% at 80 μg/ml.

**Table 4:** Cytotoxic activity of hBD-3 and analogs at different concentration.

Peptide	cytotoxic activity of hBD-3 and analogs at different concentration						
	0 μg/ml	10 μg/ml	20 μg/ml	40 μg/ml	60 μg/ml	80 μg/ml	100 μg/ml
hBD-3	0%	0%	2±2%	34±14%	39±7%	48±2%	60±3%
hBD-3Δ4	0%	0%	45±5%	61±4%	66±3%	68±6%	85±3%
hBD-3Δ7	0%	0%	0%	0%	12±2%	8±7%	11±10%
hBD-3Δ10	0%	0%	0%	0%	0%	8±1%	5±3%

## Discussion

HBD-3 and its three N-terminal deletion analogs were designed and synthesized to find new analogs that could have significant therapeutic properties. The antibacterial activities of hBD-3 and its analogs against a broad range of human Gram-negative and Gram-positive strains were compared and analyzed.

In previous studies, the effect of N-terminal residues on the antibacterial activity of hBD-3 was detected and analyzed. Scudiero et al. [15] reported that deletion of the N-terminal region of hBD-3 increases antibacterial activity. Hoover et al. [12] reported that the deletion of seven or nine N-terminal residues (hBD-3Δ8 or hBD-3Δ10) does not affect the antibacterial activity of hBD-3 against *E. coli* and *S. aureus*. In concordance with these results, hBD-3 and its N-terminal deletion analogs showed similar antibacterial activities against most of the tested bacteria, including *E. coli* and *S. aureus*. Conversely, excessive deletions of N-terminal residues can affect the antibacterial activity of hBD-3 against other bacteria. This finding may explain the lower antibacterial activity of hBD-3Δ10 against *K. pneumonia*, *S. flexneri*, and *S. sonnei*. Thus, the N-terminal region of hBD-3 has different effects on its antibacterial activity because of the diverse cell membrane structures of different bacteria. In addition, we found that the antibacterial activities of hBD-3 and its analogs, especially hBD-3Δ10, were significantly weaker against *K*. *pneumoniae* than against other bacteria. One of the possible reasons is that the capsule polysaccharide of *K*. *pneumoniae* limits the interaction of hBD-3 and its analogs with membrane targets [16]. Thus, compared with hBD-3 and its other analogs, the analog (hBD-3Δ10) with a deletion of nine residues showed significantly lower antibacterial activity against *K. pneumonia*.

Salt resistance is a factor that negatively affects the antimicrobial activity of beta-defensins. This factor depends on the net charges of beta-defensins. In the present study, although hBD-3, hBD-3Δ4, and hBD-3Δ7 have the same net charge (+11), they have different levels of salt resistance. hBD-3 was more salt resistant than hBD-3Δ7, but hBD-3Δ4 was more salt resistant than hBD-3. Yu et al. [17] found that the analog of NP-1 with clustered positive net charge has low salt resistance. Scudiero et al. [15] found that the removal of the N-terminal domain of hBD-3 presumably changes the structure into a compact conformation that would allow an efficient interaction of the peptide with the bacterial surface even at very high NaCl concentrations. However, this result does not explain why hBD-3 (45 amino acids) is more salt resistant than hBD-3Δ7 (39 amino acids). The 3D structure (such as N-terminal α-helix) of hBD-3 is another critical factor that affects its salt sensitivity. Based on the 3D structures of hBD-3, the N-terminal helix in hBD3 starts from Lys8 [17]. Thus, N-terminal residues 1 to 7 comprise a helix-capping motif, and hBD-3, hBD-3Δ4, and hBD-3Δ7 should have different lengths of helix caps. The helix-capping motifs may be responsible for the α-helix integrity at high NaCl concentrations and salt-resistant antibacterial activity of the peptides. Therefore, hBD-3Δ7 has low antibacterial activity at high NaCl concentrations because of its short helix-cap. This result can be verified by the CD result of hBD-3 and its three N-terminal deletion analogs (Fig. 3). The CD spectra of hBD-3Δ7 and hBD-3Δ10 did not show obvious α-helix structures, especially with high NaCl concentrations. Furthermore, the analog hBD-3Δ4 was more salt resistant than hBD-3 because it had a similar α-helix structure (CD result, Fig. 3) but a more compact conformation than hBD-3 with the same positive net charge. Thus, we hypothesized that both α-helix structures and positive net charge affect the salt resistance of hBD-3. And the big change in CD spectrum of hBD-3Δ10 when exposed to increasing concentration of NaCl, which showed that hBD-3Δ10 has a instability secondary structures with high NaCl concentrations. It may be one of the reasons that the antimicrobial activities of hBD-3Δ10 were lower and more salt sensitive than other peptides.

The cytotoxicity of different defensins is also an important factor affecting their antibacterial activities. In this study, we tested the cytotoxicity of hBD-3 and its analogs against vero cells. hBD-3Δ4 had no cytotoxicity on vero cells at 10 μg/ml (approximately twofold to fourfold LD_90_ against most tested bacteria). Thus, the cytotoxic side effect of hBD-3Δ4 against eukaryotic cells is still in an acceptable ratio to the cure. Similar to a previous study [18], we found that the high antibacterial activities of defensins and derivatives are often accompanied by high cytotoxicity. At high concentrations (>20 μg/ml), hBD-3Δ4 showed higher cytotoxicity than hBD-3 and the other analogs. Thus, reducing the cytotoxicity of hBD-3Δ4 may be helpful to promote its therapeutic applications.

In summary, we designed and synthesized analogs of the innate immunity peptide hBD-3. We investigated the antibacterial activities and salt resistance of hBD-3 and its analogs against a broad range of standard and clinically isolated strains. The analog hBD-3Δ4 had the highest antimicrobial activity at high NaCl concentrations. This study may contribute to the development of new antibacterial peptides against pathogens.
